# Precisely Controlled Reactive Multilayer Films with Excellent Energy Release Property for Laser-Induced Ignition

**DOI:** 10.1186/s11671-019-3124-6

**Published:** 2019-08-29

**Authors:** Wei Guo, Shimin Chang, Jinle Cao, Lizhi Wu, Ruiqi Shen, Yinghua Ye

**Affiliations:** Department of Applied Chemistry, School of Chemical Engineering, Nanjing University of Science and Technology, Nanjing, 210094 China

**Keywords:** Reactive multilayer films, Energetic flyer plates, Magnetron sputtering, Metallic oxide/aluminum, Laser ignition

## Abstract

**Abstract:**

Three types of reactive multilayer films (RMFs) were integrated to the energetic flyer plates (EFPs) by depositing TiO_2_, MnO_2_, and CuO onto aluminum films with different modulation periods using magnetron sputtering technology in this study. The effects of the laser ignition property and laser reflectivity on the RMFs and the thermal behavior of the RMFs were analyzed and compared with those of a single-layer Al film. A high-speed video, photonic Doppler velocimetry (PDV), and a thermal analysis were utilized to characterize the flame morphology, EFP velocity, and chemical thermal behavior, respectively. The surface reflectivities of the TiO_2_/Al, MnO_2_/Al, and CuO/Al layers were measured using laser reflectivity spectrometers. The results showed that RMFs with smaller modulation periods exhibited excellent laser ignition performances, and EFP with MnO_2_/Al had the best performance. These RMFs achieved flame durations of 120–220 μs, maximum flame areas of 7.523–11.476 mm^2^, and reaction areas of 0.153–0.434 mm^2^ (laser-induced with 32.20 J/cm^2^). Flyer velocities of 3972–5522 m/s were obtained in the EFPs by changing the material and modulation period of the RMFs. Furthermore, the rate of the chemical reaction and laser energy utilization were also enhanced by reducing the modulation period and using different material. This behavior was consistent with a one-dimensional nanosecond-laser-induced plasma model. The RMFs of MnO_2_/Al exhibited the highest level of energy release and promoted laser energy utilization, which could better improve the performance of laser ignition for practical application.

**Graphical Abstract:**

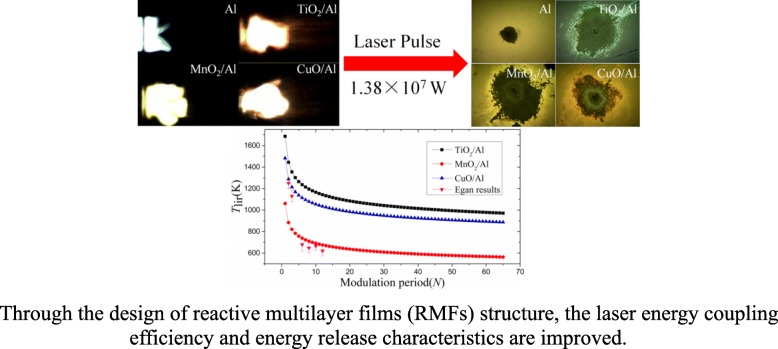

## Highlights

- Reactive multilayer films (RMFs) and energetic flyer plates were prepared by magnetron sputtering method based on TiO_2_/Al, MnO_2_/Al, and CuO/Al.
- The RMFs improves the energy conversion efficiency and ignition performance.
- Optimal addition of RMFs remarkably enhances the energy output of energetic flyer plates.

## Introduction

Reactive multilayer films (RMFs), as a typical nanoscale energetic material, are mixtures of nano-aluminum films and metal oxide films that can rapidly release chemical energy via ignition or explosion [1–3]. RMFs can react violently to release a large amount of chemical energy with a small input energy. These nano-thermite materials can be integrated into multilayer flyer energy conversion elements (as the ablative layer of a flyer plate), which can be used in a laser-driven flyer plate detonator (LDFPD) [4]. The traditional ablative layer material of a flyer plate is composed of aluminum or copper, which have high laser reflectivity (up to 80%) [4–8]. Laser energy utilization is less than 20%, resulting in wasted energy [9]. Thus, the initiation threshold of the LDFPD cannot be reduced effectively.

In order to create a safe and efficient nonlinear exchange energy (high-energy output from low-energy input) ignitor, many researchers have tried to improve the output performance by improving the laser energy utilization and reducing the initiation threshold of the LDFPD [4, 10–12]. RMFs are a type of thermite nano-energetic multilayer film that has high energetic density and high-energy release rate [13–17]. Furthermore, the preparation of RMFs is simple, and the structure is controllable. The output capability of the LDFPD could be improved if better materials and structures of RMFs were applied to the ablation layer of the flyer plate. An LDFPD system with RMFs has a safer and more reliable initiation than those of an electric explosive device, such as a semiconductor bridge (SCB) or an exploding foil initiator (EFI), especially in a strong electromagnetic environment [17–19]. Therefore, RMFs could be applied in the LDFPD as the ablation layer of the flyer plate to provide higher output energy and enhance the conversion efficiency of the laser.

Based on the advantages listed above, RMFs are an excellent nano-energetic structure material for integration into the LDFPD system, which could increase the efficient nonlinear exchange energy rate of the flyer plate [20–22]. Therefore, the initiation threshold of the LDFPD could be reduced.

In this study, three types of RMFs were integrated to the energetic flyer plates (EFPs, the term energetic flyer plate is used to describe the entire flyer plate assembly, including the glass substrate, RMF, Al_2_O_3_ thermal insulation layer, and Al flyer layer) by depositing different materials (TiO_2_, MnO_2,_ and CuO) onto aluminum films with different modulation periods using a magnetron sputtering technology, allowing precise control of the nano-structure of the RMFs. Through the optimization of the layered material (TiO_2_/Al, MnO_2_/Al, and CuO/Al) and structure, ablation layer systems of the flyer plates with higher energy output and higher chemical reaction activity than those of a traditional single-layer Al film were obtained. The ignition performances of various RMFs, including the flame property, laser reflectivity, and thermal behavior of the RMFs, were systematically studied using different layered structures. Moreover, the induced temperature (*T*_lir_) of the MnO_2_/Al, CuO/Al, and TiO_2_/Al [22] was calculated to study their energy release characteristics.

## Methods

### Fabrication of the RMFs and EFPs

The composite films were fabricated using a magnetron sputtering technology. K9 glass (Φ 50 mm × H 2 mm and Φ 5 mm × H 2 mm, Shanghai Jiujing Optoelectronic Technology Co. Ltd) was used to create the RMFs and EFPs. A magnetron sputter coating apparatus was used for the rounded RMFs because the sputtering target material fabricated thick film graphics with a high accuracy and homogeneity. The K9 glass was cleaned with pure acetone, ethanol, and deionized water at 45 °C in an ultrasonic bath for 30 min and then placed in an oven for 5 h at 50 °C.

As stated above, thermite RMFs, consisting of alternating nano-layers of metal oxide and aluminum [16], are a new type of nano-thermite with a high application value. To obtain RMFs with different thickness, a radiofrequency (RF) magnetron sputtering technique was used. Magnetron sputtering uses a low temperature and high speed relative to other preparation methods, meeting the preparation requirements of the nanoscale multiple alternate layers and providing an accurate control layer thickness in a composite membrane.

**Fig. 1:**
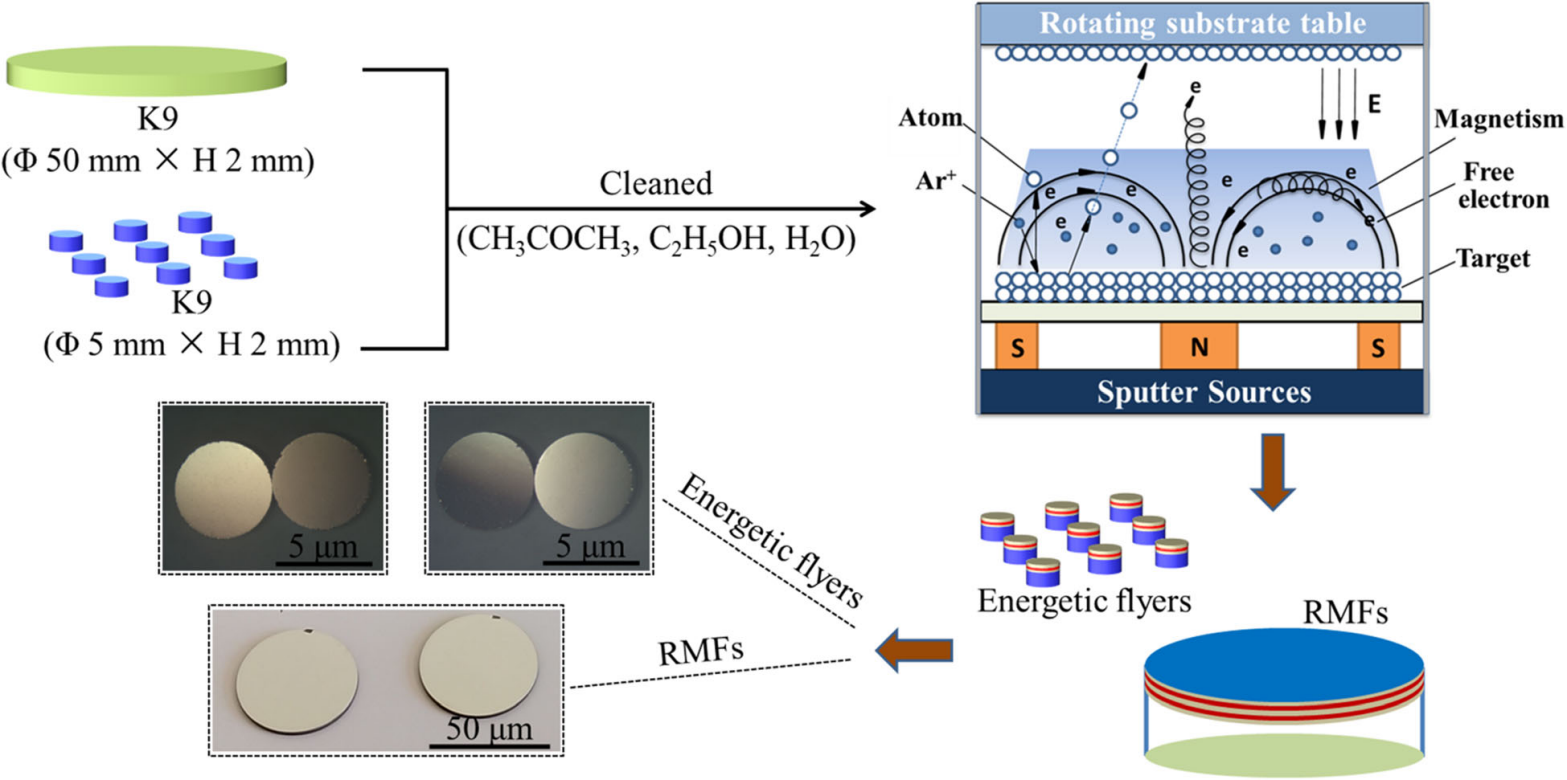
The depositing formation process of RMFs and EFPs

Figure 1 illustrates the accurate depositing formation of RMFs and EFPs. The TiO_2_ (purity > 99.99%), MnO_2_ (purity > 99.99%), CuO (purity > 99.99%), Al_2_O_3_ (purity > 99.99%), and Al (purity > 99.999%) targets (Nanchang guocaikeji co. LTD) were sputtered using RF at 229 W, 244 W, 245 W, 234 W, and 204 W, respectively. The base pressure of the vacuum chamber was 3.0 × 10^−3^ Pa with a working pressure of 0.4 Pa (gas flux of 1.8 × 10^−3^ m^3^/h) of argon (99.999%). A rotating substrate tray was used to implement multiple alternate deposition times to adjust the thickness of the three different types of RMFs. Here, 132- and 400-nm thicknesses were selected for the RMF modulation periods, and the total thickness was approximately 400 nm. Then, the round RMFs were covered with sputtered Al_2_O_3_ (0.6 μm) and Al (3 μm) films for the EFP. In order to facilitate the identification, the various types of RMFs with different modulation periods were denoted as TiO_2_/Al-132 nm, MnO_2_/Al-132 nm, CuO/Al-132 nm, TiO_2_/Al-400 nm, MnO_2_/Al-400 nm, and CuO/Al-400 nm, and the various types of EFPs were denoted by (TiO_2_-Al)_III_-Al_2_O_3_-Al, (MnO_2_-Al)_III_-Al_2_O_3_-Al, (CuO-Al)_III_-Al_2_O_3_-Al, (TiO_2_/Al)_I_-Al_2_O_3_-Al, (MnO_2_/Al)_I_- Al_2_O_3_-Al, and (CuO/Al)_I_-Al_2_O_3_-Al.

### Characterization Methods

The composition and microstructure morphology of the multilayers were determined using field emission scanning electron microscopy (FESEM, S-4800 II) and X-ray photoelectron spectroscopy (XPS, PHI QUANTERA II SXM). To investigate the ignition characteristics of the various RMFs, a Q-switched Nd:YAG (1064 nm, 6.5 ns) laser system was adopted with a high-power-density laser on the surface of the RMFs. In the experiment, a Gaussian short pulses laser (6.5 ns) was produced by the laser system, and a convex lens focused the beam at 0.6 mm, and then, the surface of the RMFs was irradiated. The laser-induced process of the RMFs was recorded using a high-speed camera (HG-100K) that captured 50,000 frames per second. The laser provided a synchronized trigger signal to a high-speed video. The flight process was characterized using a photonic Doppler velocimetry (PDV) system.

Surface reflectivity measurements of the RMFs were performed using laser reflectivity spectrometers (AvaSpec-NIR256-1.7). When the laser light reached the surface of the RMFs, the laser was reflected inside the integrating sphere numerous times. Therefore, the laser light intensity of every point of the sphere’s internal surface was the same with a uniform distribution. The reflected light of the laser was measured by the spectrometer, and the synchronized reflectivity was calculated by computer software. In order to examine the temperature of the exothermic reaction in the TiO_2_/Al RMFs, a K9 glass wafer (Φ 50 mm × H 2 mm) was coated with a thin layer of photoresist sedimentation. The TiO_2_/Al RMFs could separate and peel off after the photoresist was dissolved. Then, a differential thermal analysis (DTA, TA Instruments SDT600) was conducted from 30 to 1000 °C under N_2_ flow. The DTA tests were used to investigate the activation energy of the exothermic reaction at a heating rate of 5 to 20 °C min^−1^.

## Results and Discussion

### Layer Structure and Morphological Analysis of the Various RMFs

**Fig. 2:**
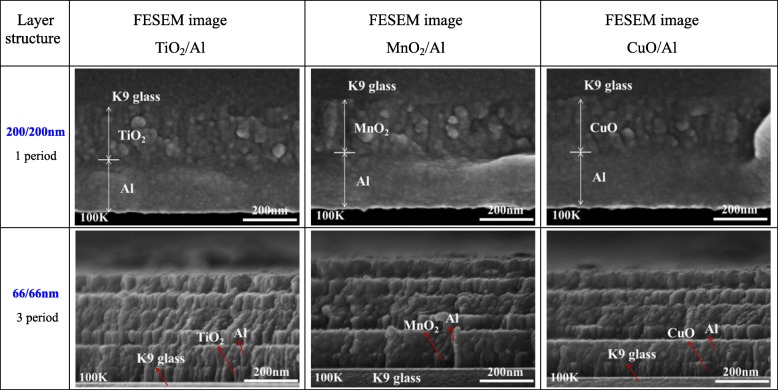
The cross-sectional FESEM images of the different integrated RMFs on the K9 glass

**Fig. 3:**
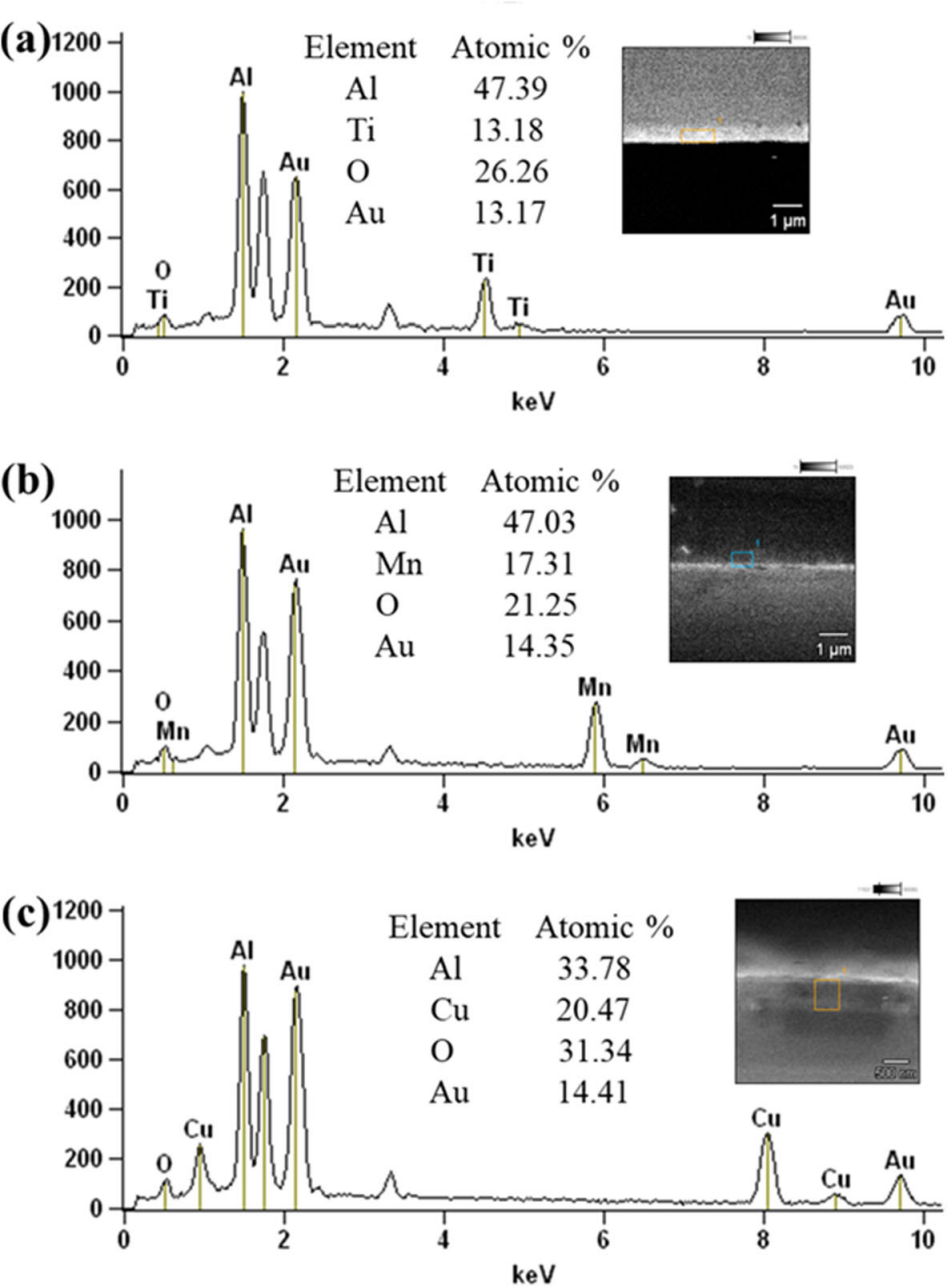
EDS patterns on the RMFs **a** TiO_2_/Al, **b** MnO_2_/Al, and **c** CuO/Al

**Fig. 4:**
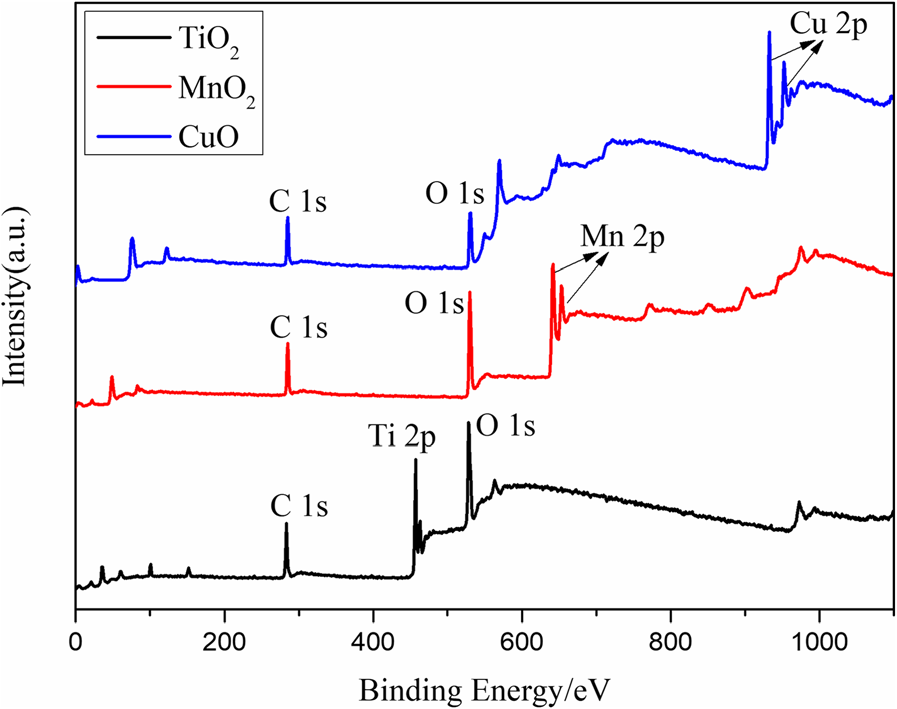
XPS spectrum of TiO_2_, MnO_2_, and CuO samples

Figure 2 shows the FESEM images of the different cross-sectional areas of the various RMFs. TiO_2_, MnO_2_, CuO, and Al layers were alternately deposited onto the surface of the K9 glass using the precision of the magnetron sputtering technology. The modulation periods of each RMF were 400 nm (TiO_2_/Al, MnO_2_/Al, CuO/Al: 200/200 nm, 1 period) and 132 nm (TiO_2_/Al, MnO_2_/Al, CuO/Al: 66/66 nm, 3 periods). In addition, the heat flow and chemical reaction velocity were changed by controlling the modulation period of the various RMFs [23, 24]. Therefore, the laser-induced plasma ignition performance of the RMFs could be tuned by varying the layered structure of the RMFs. As shown in Fig. 2, the cross-sectional FESEM images of the RMFs showed that the TiO_2_, MnO_2_, CuO, and Al layers had distinct boundary layer structure lines. The EDS analysis was performed using different RMFs deposited on the K9 glass substrate. The EDS patterns of Ti, Mn, Cu, O, and Al (Fig. 3) confirmed the presence of each expected material in the three types of RMFs. XPS analysis was performed using RMFs deposited on K9 glass substrate, as shown in Fig. 4. The XPS analysis of RMFs revealed the presence of TiO_2_ [22], MnO_2_, and CuO.

### Laser-Induced Energy Release Property for the RMFs and EFPs

**Fig. 5:**
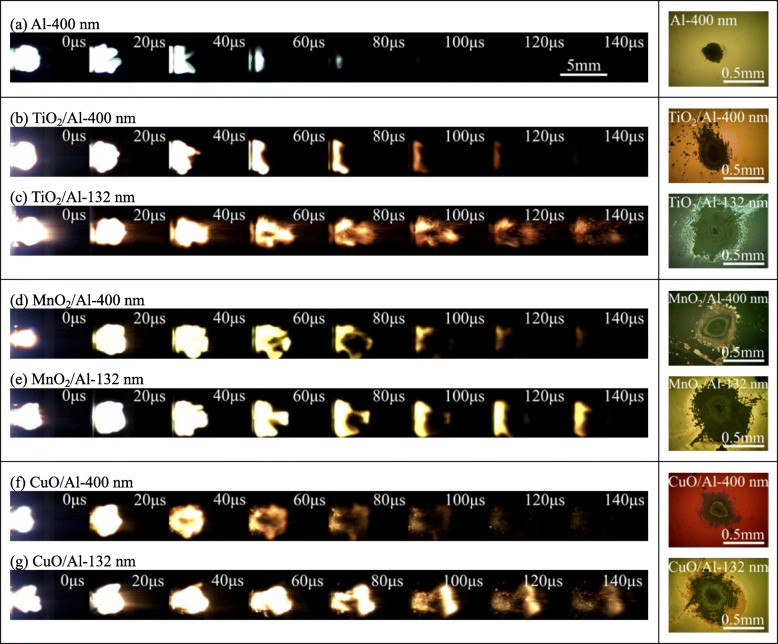
Laser-induced processes of the single-layer Al film and various RMFs and their microscope images after ignition. **a** Al-400 nm. **b** TiO_2_/Al-400 nm. **c** TiO_2_/Al-132 nm. **d** MnO_2_/Al-400 nm. **e** MnO_2_/Al-132 nm. **f** CuO/Al-400 nm. **g** CuO/Al-132 nm

The laser-induced energy release property of the RMF was characterized by high-speed photography. The energy release properties of the RMFs with different modulation periods were analyzed using high-speed video. For comparison, the same experimental study was carried out on a single-layer Al (400 nm) film. Figure 5 shows the laser-induced process of TiO_2_/Al, MnO_2_/Al, CuO/Al, and single-layer Al. The ignition processes were qualitatively measured and recorded using a high-speed camera induced at 32.20 J/cm^2^. The frames on the right side are the optical microscope images of various films after burning. The most intensive and luminous combustion reaction was observed for RMFs-132 nm (TiO_2_/Al-132 nm, MnO_2_/Al-132 nm, and CuO/Al-132 nm), indicating that the reactivity and combustion reaction of the RMFs were significantly enhanced with the reduction of the modulation period. Furthermore, the burning flame area of the MnO_2_/Al-132 nm was the largest while that of TiO_2_/Al-132 nm was the smallest.

Table 1 lists the ignition properties of single-layer Al, RMFs-400 nm (TiO_2_/Al-400 nm, MnO_2_/Al-400 nm, and CuO/Al-400 nm), and RMFs-132 nm induced at 32.20 J/cm^2^, including their reaction duration, maximum flame length, width, and area of the films. Their ignition durations were recorded at 80, 120, 180, 160, 220, 140, and 200 μs, respectively, and their maximum flame areas were measured at 6.945, 7.523, 9.835, 8.760, 11.476, 8.190, and 10.475 mm^2^, respectively. In addition, the reaction areas of various RMFs were measured from the right side frames in Fig. 5. The ablative areas were measured at 0.055, 0.153, 0.368, 0.239, 0.434, 0.191, and 0.403 mm^2^. The reaction areas of the RMFs-132 nm sample were larger. Either MnO_2_/Al-400 nm or MnO_2_/Al-132 nm, they had larger reaction areas than the other corresponding films, which also indicated the higher reactivity.

**Table 1:** Laser-induced properties of the single-layer Al film and various RMFs induced at 32.20 J/cm^2^

Sample	Ignition duration (μs)	Maximum flame length (mm)	Maximum flame width (mm)	Maximum flame area (mm^2^)	Reaction area (mm^2^)
Al-400 nm	80	2.90	3.00	6.945	0.055
TiO_2_/Al-400 nm	120	3.00	3.45	7.523	0.153
TiO_2_/Al-132 nm	180	4.05	3.33	9.835	0.368
MnO_2_/Al-400 nm	160	4.25	2.85	8.760	0.239
MnO_2_/Al-132 nm	220	3.95	3.83	11.476	0.434
CuO/Al-400 nm	140	3.75	3.50	8.190	0.191
CuO/Al-132 nm	200	4.50	3.25	10.475	0.403

**Fig. 6:**
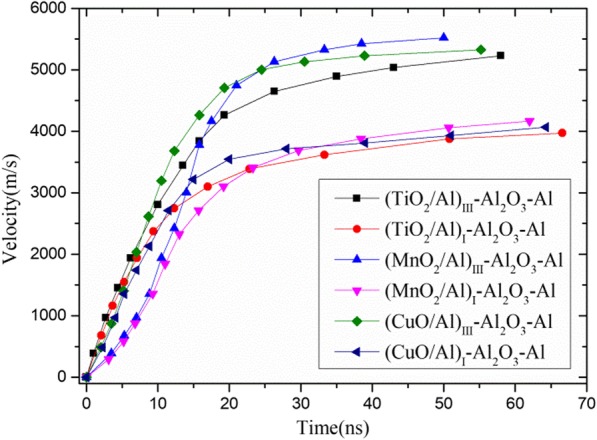
Velocity-time curves reconstructed from PDV signal induced at 32.20 J/cm^2^

Figure 6 shows a series of flyer velocity-time curves induced at 32.20 J/cm^2^. For the same laser energy, the behavior of the EFP (the EFP is composed of the K9 glass substrate, laser energy absorption RMF layer, Al_2_O_3_ thermal insulation layer, and Al flyer impact layer) [22] was similar in principle; however, the results were different. Generally, the peak velocity increased as the modulation period decreased. Terminal flyer velocities of 5231 ((TiO_2_/Al)_III_-Al_2_O_3_-Al), 3972 ((TiO_2_/Al)_I_-Al_2_O_3_-Al), 5522 ((MnO_2_/Al)_III_-Al_2_O_3_-Al), 4166 ((MnO_2_/Al)_I_-Al_2_O_3_-Al), 5328 ((CuO/Al)_III_-Al_2_O_3_-Al), and 4066 ((CuO/Al)_I_-Al_2_O_3_-Al) m/s were observed. Although the EFP with RMF was induced more completely, there was more contribution to the pressure generated by the modulation period of 132 nm. Furthermore, the EFPs with MnO_2_/Al were better for plasma than the other corresponding EFPs. When the pressure to push the flyer layer forward increased, the flyer velocity increased. In addition, the EFP was accelerated quickly, and the time required to reach the peak velocity was shorter, since the RMF was intensely induced. The progress of the EFP flight could be divided into three phases. In the first phase, at the beginning of laser irradiation as the plasma expanded rapidly, and the flyer layer was then cut out by the edges of the barrel (T10 steel, with a thickness of 0.6 mm, an outside diameter of 7.0 mm, and an inner diameter of 0.7 mm), the flyer velocity rapid increased. As time increased, the slope of the flyer velocity was reduced in the second phase, resulting in a sharp decrease in pressure and the existence of air. In the last phase, a plateau occurred because a balance between the pressure and air resistance was reached. However, there was a slow velocity of the (MnO_2_/Al)_III_-Al_2_O_3_-Al and (MnO_2_/Al)_I_-Al_2_O_3_-Al EFPs at the beginning of the plasma explosion and a highest velocity at the last phase. Nonetheless, all the EFPs reached a peak velocity of 80% within 20 ns.

### Laser Reflectivity of the Various RMFs

**Fig. 7:**
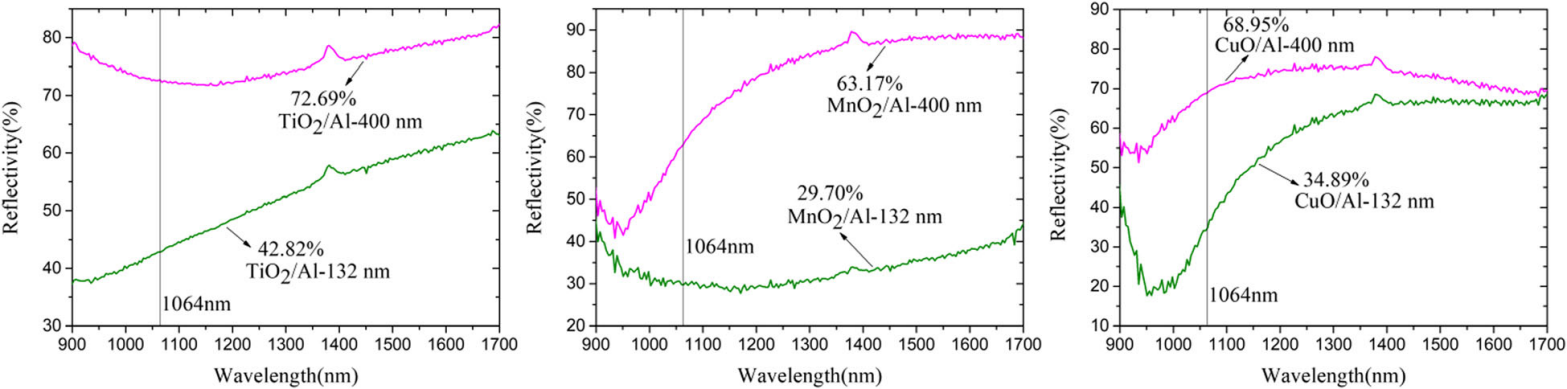
The reflectivity of various RMFs

In order to verify the influence of the material and modulation period on the laser conversion efficiency of the RMFs, the laser reflectivity was tested. Figure 7 shows the reflectivity of the various RMFs under the laser wavelengths of 900 to 1700 nm. The reflectivities of TiO_2_/Al-132 nm [22], MnO_2_/Al-132 nm, and CuO/Al-132 nm were 29.87%, 33.47%, and 34.06% lower than those of TiO_2_/Al-400 nm [22], MnO_2_/Al-400 nm, and CuO/Al-400 nm at 1064 nm, respectively. The addition of the nano-multilayer films of RMFs-132 nm decreased the reflectivity. Thus, more laser pulse energy could be used in the ablation layer of the RMFs to generate a flame. Moreover, the MnO_2_/Al-132 nm had the lowest reflectivity and absorbed more laser energy, producing a larger reaction area and higher flyer velocity.

### Thermal Analysis of Various RMFs

**Fig. 8:**
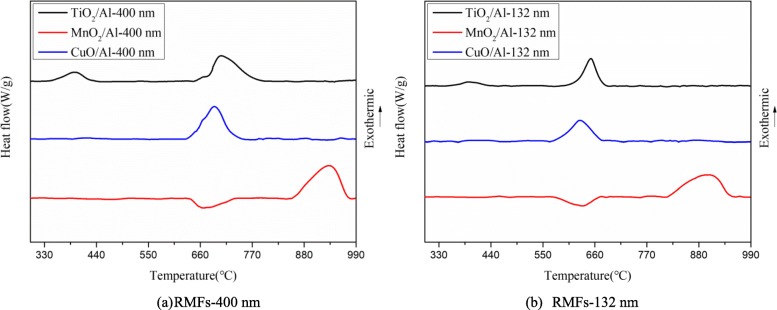
**a**, **b** DTA plots of RMFs with two different modulation periods at heating rate of 20 °C min^−1^

Thermal behavior is commonly used to evaluate the reactivity and ignition sensitivity of nano-thermite [25]. To analyze the laser-induced ignition mechanism of the RMFs, the reaction activation energy of the three types of RMFs with different heating rates were analyzed and calculated. The heating rate of the DTA analysis was set at 20 °C min^−1^ in order to determine the influence of the different modulation periods of the RMFs chemical exothermic reaction temperature, as shown in Fig. 8. The remarkable exothermic peaks of the TiO_2_/Al, MnO_2_/Al, and CuO/Al with modulation periods of 132 nm were observed at 652.19, 899.17, and 624.98 °C, respectively. For the thicker modulation period of TiO_2_/Al-400 nm, MnO_2_/Al-400 nm, and CuO/Al-400 nm, there were obvious exothermic peaks at 706.16, 933.62, and 685.96 °C, respectively. The smaller exothermic peaks were shown at 392.18 and 395.74 °C, corresponding to TiO_2_/Al-132 nm and TiO_2_/Al-400 nm, respectively. For the MnO_2_/Al-132 nm and MnO_2_/Al-400 nm, there were obvious endothermic peaks at 634.24 and 666.71 °C, respectively. The peak temperature of the main exothermic reaction decreased as the modulation periods of the RMFs decreased. This suggested that the smaller modulation periods of the RMFs had a higher reactivity owing to the increase of the interfacial area of the contact interface (between metallic oxide and Al films) [26–28]. Meanwhile, the heat release increased as the modulation periods of the RMFs increased. This was because the increasing area of the contact interface (between metallic oxide and Al films) made the oxygen of the metallic oxide layer diffuse into the Al layer spontaneously and react with the Al atoms, resulting in a misuse of energy [2, 16, 24, 29–31]. For the RMFs of TiO_2_/Al, the first exothermic peak corresponded to the solid-solid reaction between TiO_2_ and Al before Al melting, and the second peak represented the liquid-solid reaction between the melted Al and TiO_2_. The main heat releases of TiO_2_/Al-132 nm and TiO_2_/Al-400 nm at a heating rate of 20 °C·min^−1^ calculated by Universal Analysis 2000 software were 694.1 and 911.3 J/g, respectively, supporting its potential application as an RMF. For the RMFs of MnO_2_/Al, there was an endothermic peak at around 660 °C, which was generated by the melting of aluminum. After melting of Al film, the melted Al reacted with a MnO_2_ beneath the MnO_2_ nano-films at an onset temperature of about 830 °C. This exothermal reaction occurred by a liquid-solid diffusion mechanism [32, 33]. The heat releases of MnO_2_/Al-132 nm and MnO_2_/Al-400 nm at a heating rate of 20 °C min^−1^ were 1241.0 and 1659.0 J/g, respectively. CuO/Al-132 nm and CuO/Al-400 nm have an exothermic peak at 630.31 and 689.78 °C, and the heat releases were 823.1 and 1091.0 J/g, respectively.

**Fig. 9:**
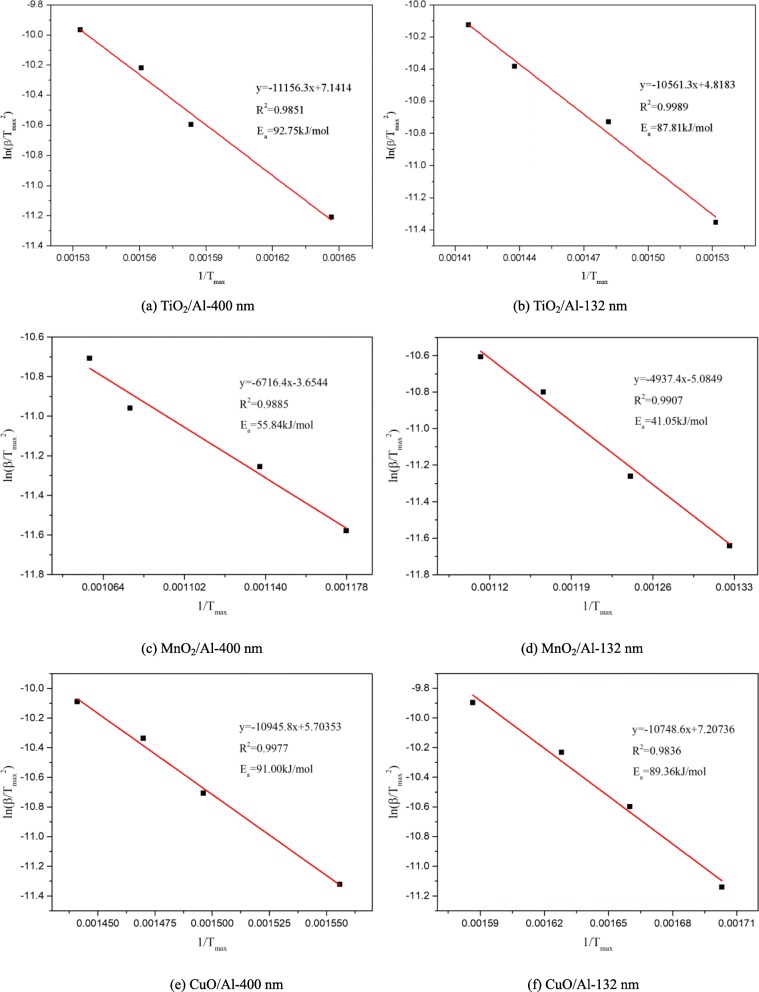
The activation energy was calculated using the Kissinger method. **a** TiO_2_/Al-400 nm, **b** TiO_2_/Al-132 nm, **c** MnO_2_/Al-400 nm, **d** MnO_2_/Al-132 nm, **e** CuO/Al-400 nm, and **f** CuO/Al-132 nm (here, the *β* is the heating rate of DTA analysis, and *T*_max_ is the peak temperature of the exothermic reaction)

The Kissinger method was used to obtain the RMF reaction activation energy (*E*_a_) of the exothermic peak [25]. The DTA data of various RMFs were fitted using the Kissinger method at different heating rates of 5 to 20 °C min^−1^, as shown in Fig. 9. The main reaction exothermic peaks greater than 600 °C in the DTA curves were selected, and the reaction activation energies were estimated as 92.75 kJ/mol (TiO_2_/Al-132 nm) [22], 87.81 kJ/mol (TiO_2_/Al-400 nm) [22], 41.05 kJ/mol (MnO_2_/Al-132 nm), 55.84 kJ/mol (MnO_2_/Al-400 nm), 89.36 kJ/mol CuO/Al-132 nm, and 91.00 kJ/mol (CuO/Al-400 nm). The DTA analysis shows that the MnO_2_/Al film has the lowest reaction activation energy, followed by TiO_2_/Al and CuO/Al film, and the modulation periods of RMFs had minimal influence on the exothermic activation energy.

### Laser-Induced Ignition Model for the RMFs

The RMF modulation period had a straightforward effect on the laser-induced process. A laser ignition model combining the thermal conduction and mass-diffusion reaction [2, 34] was proposed to study the effect of the modulation period of the RMFs on the laser ignition. High-temperature plasma was produced by the laser ignited RMFs. Therefore, the chemical reaction of the RMFs further increased the temperature. The thermal equilibrium ablation process and mass-diffusion reaction mechanism were analyzed by the equations of state [2, 16, 34]. The laser-induced temperature (*T*_lir_) of the RMFs is given as follows:

1 $$ \frac{\mathrm{\partial T}}{\mathrm{\partial t}}=\frac{1}{{\mathrm{C}}_{\mathrm{v}}}\left[\frac{\partial }{\mathrm{\partial x}}k\frac{\mathrm{\partial T}}{\mathrm{\partial x}}+\mathrm{A}\left(\mathrm{x},\mathrm{t}\right)\right] $$

2 $$ {\mathrm{T}}_{\mathrm{lir}}=\frac{-{\mathrm{E}}_{\mathrm{a}}}{\mathrm{R}}{\left\{\ln \left[\frac{\frac{1}{{\mathrm{C}}_{\mathrm{v}}}\left[\frac{\partial }{\mathrm{\partial x}}k\frac{\mathrm{\partial T}}{\mathrm{\partial x}}+\mathrm{A}\left(\mathrm{x},\mathrm{t}\right)\right]{\mathrm{C}}_{\mathrm{p}}}{\mathrm{nA}\Delta  {\mathrm{H}}_{\mathrm{rnx}}\frac{\mathrm{\partial C}}{\mathrm{\partial x}}{\mathrm{D}}_0}\right]\right\}}^{-1} $$

The parameter *T* is the laser-induced plasma temperature, and *C*_v_ is the sum heat capacities of the ablative material ion and electron subsystems. The parameter *k* and *A*(*x*,*t*) are the thermal conductivity and energy source from the laser [35–38], respectively. The parameter *C*_p_ is the heat capacity, and *n* is the number of interfaces (*n* = 2 *N* − 1) (*N* is the number of modulation periods). *A* (m^2^) is the reaction surface area; *∆H*_rnx_ (J/mol) is the energy released of the reaction. The parameter $\frac{\partial C}{\partial x}$ (mol/m^4^) is the concentration of 1.250 × 10^13^ mol/m^4^ (TiO_2_/Al) [22], 1.446 × 10^13^ mol/m^4^ (MnO_2_/Al), and 2.074 × 10^13^ mol/m^4^ (CuO/Al). The parameter *D*_0_ was 2.9 × 10^−10^ m^2^/s calculated by Egan [2]. The parameter *E*_a_ is the activation energy of the material.

**Fig. 10:**
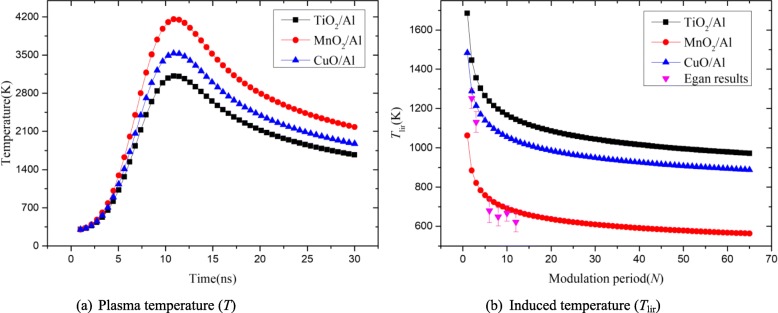
Relationship of modeled **a** laser-induced plasma temperature (*T*) over times and **b** induced temperature (*T*_lir_) with number of modulation periods of the RMFs

The RMFs were irradiated by a 1064-nm laser for 6.5 ns, and the initial temperature (*T*_0_) was 300 K. For the change of materials (TiO_2_/Al, MnO_2_/Al, and CuO/Al) and number of modulation periods (*N*), the induced temperature and number of modulation periods are shown in Fig. 10a and b, respectively.

Figure 10a shows the one-dimensional distribution of the plasma temperature (*T*) under laser irradiation. This is a typical phenomenon caused by nanosecond laser ablation. When time was in the range of approximately 0–10 ns, the temperature of the RMFs increased rapidly. Then, the temperature decreased. For different materials, it can be found that the *T* of MnO_2_/Al was the highest, followed by CuO/Al and TiO_2_/Al. Figure 10b shows the one-dimensional distribution of the *T*_lir_ with different materials. When *N* was augmented, the *T*_lir_ of the RMFs decreased rapidly (*N* < 16) and then was saturated (*N* ≥ 16). The modeled induced temperature shows that the MnO_2_/Al film has the lowest *T*_lir_, and the modulation periods of RMFs had analogical influence on the *T*_lir_. By comparing the *T*_lir_ in reference (tested by Egan) [2], it can be found that they had similar rule, namely, the temperature (*T*_lir_) decreased with the increase of modulation period, which was also instructive for the model. These results indicated that the chemical reactivity could be controlled with different materials and modulation periods, which were consistent with the change of the ignition performance of the RMFs.

The above results show that RMFs with MnO_2_/Al film and a smaller modulation period have a higher reactivity and lower *T*_lir_. Therefore, the MnO_2_/Al-132 nm had a higher reaction rate. By choosing different materials and comparing different structures, we obtained an EFP ((MnO2-Al)_III_-Al2O3-Al) with better performance than that in the reference [22]. It can be found in the thermal behavior and laser ignition of the RMFs that the reactivity and ignition sensitivity of the flame for the MnO_2_/Al nm film was more superior than that for the TiO_2_/Al and CuO/Al film, which caused the flame mass to be distributed over a larger volume. Furthermore, MnO_2_/Al-132 nm film not only has the lowest reaction activation energy and high heat release, but also has the lowest laser reflectivity. This explained the high energy conversion efficiency and energy release rate of MnO_2_/Al-132 nm film in laser ignition. These excellent properties determined that the MnO_2_/Al-132 nm film exhibited excellent performances with a maximum flame area, reaction area, and flyer velocity.

## Conclusions

In this study, three types of RMFs (TiO_2_/Al, MnO_2_/Al, and CuO/Al) and EFPs with two different modulation periods (i.e., 400 and 132 nm) were prepared by magnetron sputtering. Using FESEM, the cross-section showed the structure of the RMFs, and the layers were tightly combined. Using high-speed video and spectrometers to measure the ignition flame and laser reflectivity of thin films, the RMFs with a modulation period of 132 nm had the maximum ignition flame areas and minimum reflectivities. Furthermore, the MnO_2_/Al-132 nm had the highest reaction area and the lowest reflectivity. Specifically, the laser-induced energy release performance of the RMFs was significantly improved, compared with the single-layer Al film. Among them, the RMF of MnO_2_/Al was the best material to improve the energy release performance. From the velocity curve of the EFP, the flyer plate of the MnO_2_/Al with three modulation periods also showed a higher velocity. The energy coupling efficiency of the laser and thin film could be easily tuned by controlling the material and modulation period of the RMFs, therefore changing the ignition performance of the RMFs. Moreover, this behavior was consistent with a laser-induced ignition model. The RMF preparation detailed in this article exhibited a controlled ignition performance, and the RMF of particular materials could potentially be used in high safety ignition under a complex electromagnetic environment, especially in optimizing the launch and impact characteristics of laser-driven flyer plate.

## Data Availability

Authors declare that the materials, data, and associated protocols are available to the readers, and all the data used for the analysis are included in this article.

## Abbreviations

EFPs: Energetic flyer plates
LDFPD: Laser-driven flyer plate detonator
PDV: Photonic Doppler velocimetry
RMFs: Reactive multilayer films
